# Supplementary material for: MiR-125b-2 Knockout in Testis Is Associated with Targeting to the PAP Gene, Mitochondrial Copy Number, and Impaired Sperm Quality
Source: Int J Mol Sci. 2019 Jan 3;20(1):148. doi: 10.3390/ijms20010148 (PMC6337273; doi:10.3390/ijms20010148)
Supplement: Supplementary file 1 [file ijms-20-00148-s001.pdf]

Supplementary Materials

Table S1. Characteristics of the reads from 2 different mouse testis. A total of two samples were analyzed, which included the mixing of three testes for each condition (WT and KO).

Sample	Raw Data		Valid Data		Valid Ratio%	Q20%	Q30%	GC content%
	Read	Base	Read	Base	Reads			
WT_G	56391004	8.46G	55911230	8.39G	99.15	99.66	94.90	48
KO_G	48969660	7.35G	48561786	7.28G	99.17	99.57	94.03	48

Table S2. Ten genes with the most significant differences in up-regulation in KO mouse testis.

Gene name	Description
mt-Nd2	mitochondrial encoded NADH dehydrogenase 2
Actrt2	actin-related protein T2
Kif2b	kinesin family member 2B
Gtsf1l	gametocyte specific factor 1-like
Gm5617	predicted gene 5617
Cetn1	centrin 1
Gm9795	predicted pseudogene 9795
Papolb	poly (A) polymerase beta (testis specific)
4930407I10Rik	RIKEN cDNA 4930407I10 gene
Hypm	huntingtin interacting protein M

Table S3. Ten genes with the most significant differences in down-regulation in KO mouse testis.

Gene name	Description
Smcp	sperm mitochondria-associated cysteine-rich protein
mt-Cytb	mitochondrial encoded cytochrome b
Hils1	histone H1-like protein in spermatids 1
Gk2	glycerol kinase 2
Eid3	EP300 interacting inhibitor of differentiation 3
Hoga1;4933411K16Rik	4-hydroxy-2-oxoglutarate aldolase 1 Symbol; Acc:MGI:1914015]
Prm3	protamine 3
1700003E24Rik	RIKEN cDNA 1700003E24 gene
4930403D09Rik	RIKEN cDNA 4930403D09 gene

Table S4. siRNA oligos target to PAP gene were synthesized.

Product name	Target sequence
PAP siRNA1	CCACCTAAGCCTACAATGA
PAP siRNA2	CCCATAGAAAGCTCAGGAA
PAP siRNA3	GAAATACAGCAACGAACAT

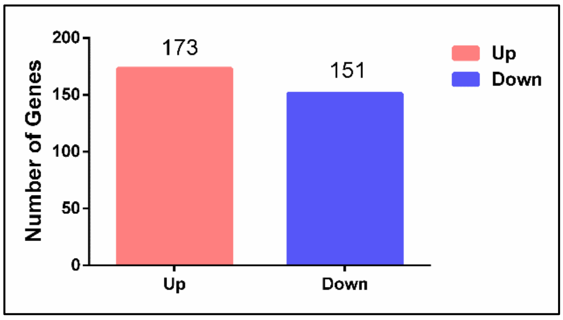

Figure S1. Up-regulated and down-regulated genes number in testicular transcriptome between KO mice and WT mice.

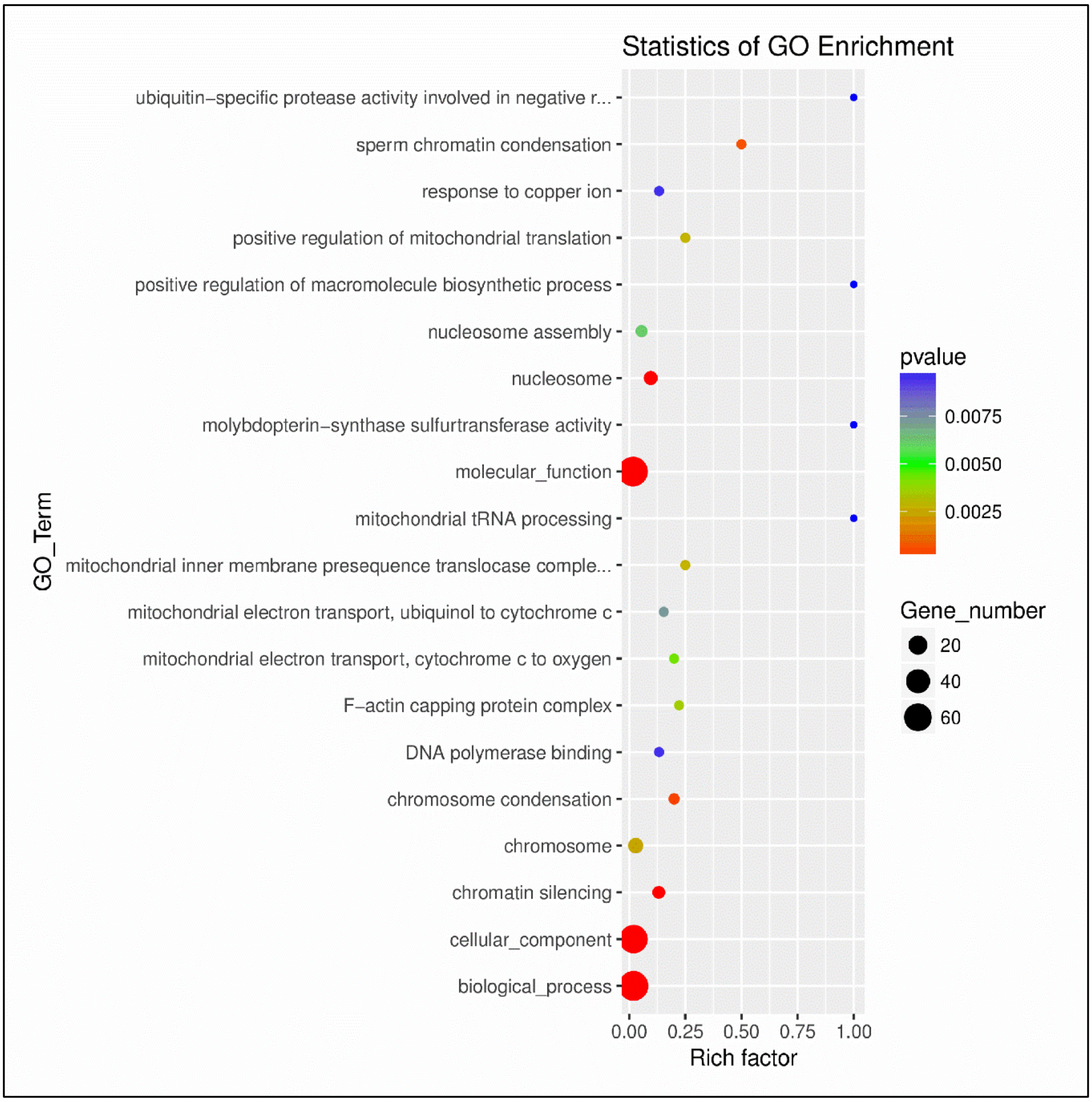

Figure S2. Analysis of biological functions of GO-enriched genes from KO and WT.

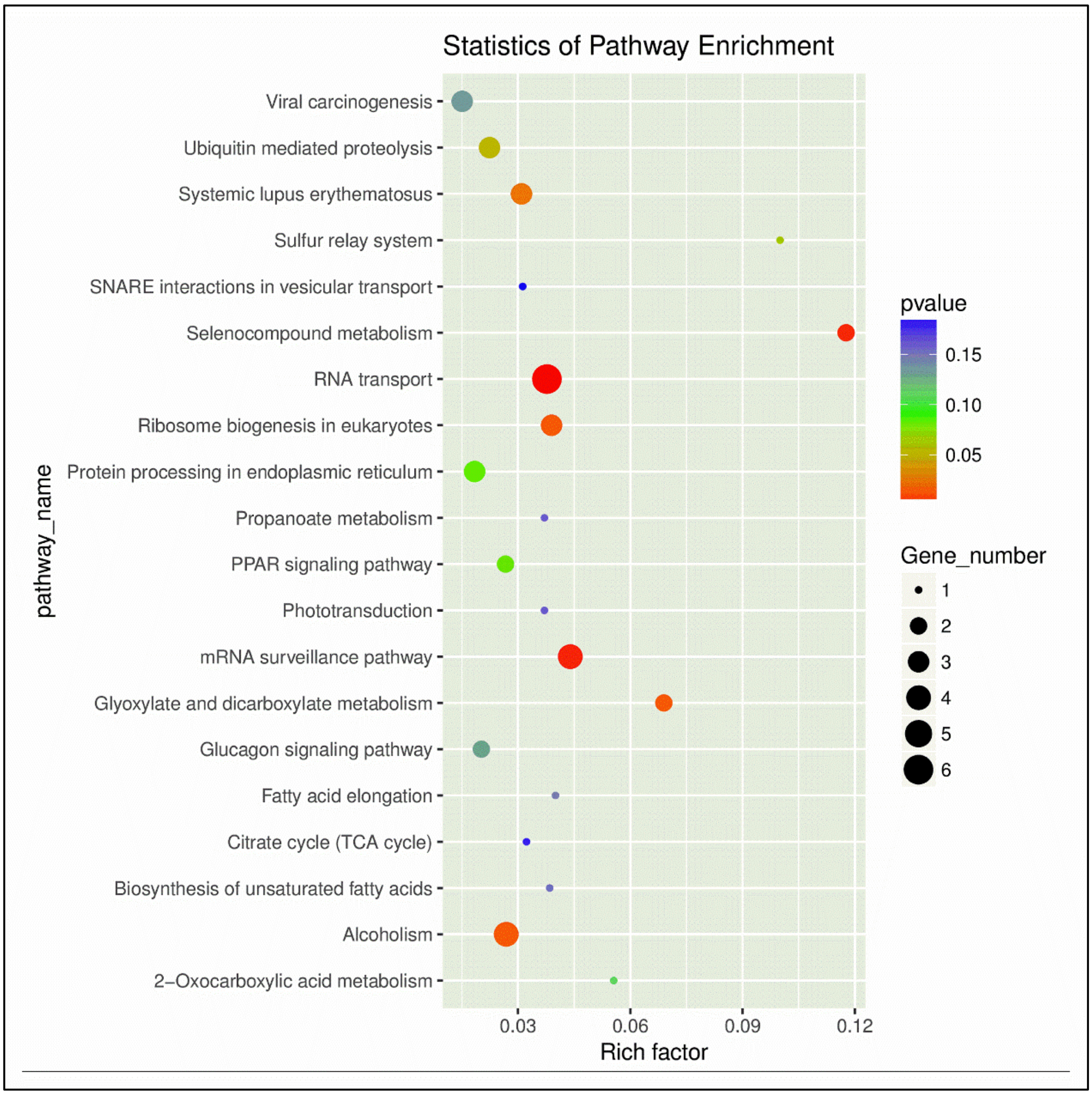

Figure S3. KEGG pathway enrichment analysis of differentially expressed genes from KO and WT.

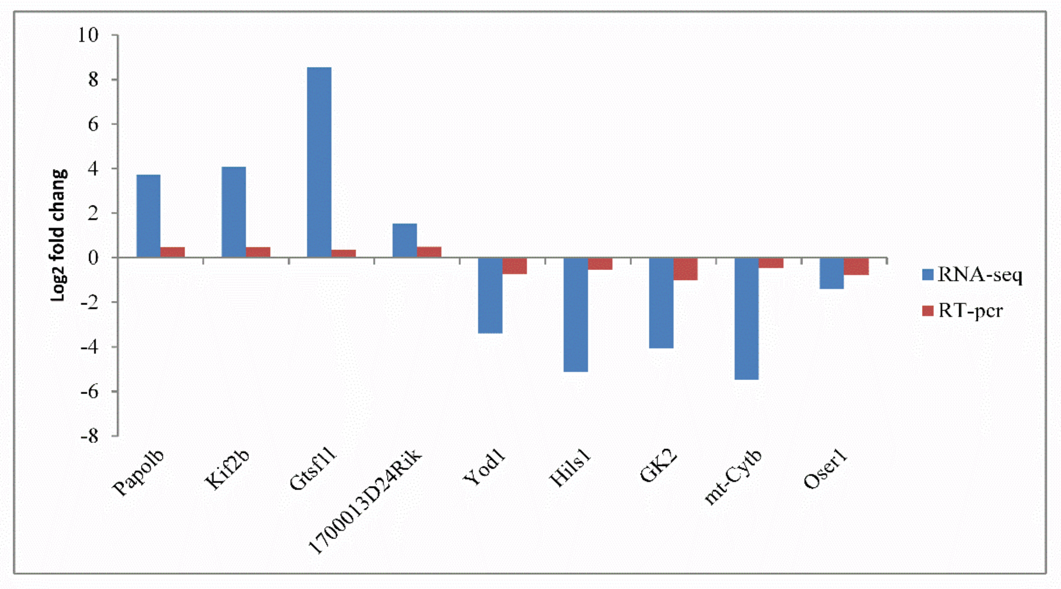

Figure S4. Validation of relative gene expression obtained from RNA-seq by qRT-PCR (n=6). Relative expression values of qRT-PCR are presented as an average.

Table S5. The primers of RT-PCR were synthesized.

Primer names	Sequenceamesof R
ND1-F	ATTGCACCTACCCTATCACTCAC
ND1-R	ACGGCTCGTAAAGCTCCGAA
ND4-F	ACAGCCTGATTACTGCCACT
ND4-R	GTTTGGTTCCCTCATCGGGT
ATPase 6 - F	CAAACAAATAATGCTAATCCACACACC
ATPase 6 - R	GCTGTAAGCCGGACTGCTAATG
Mit-1000 - F	CGATAAACCCCGCTCTACCT
Mit-1000 - R	AGCCCATTTCTTCCCATTTC
mt-Cytb - F	AACATACGAAAAACACACCCATT
mt-Cytb - R	AGTGTATGGCTAAGAAAAGACCTG
COX2-F	AACCGAGTCGTTCTGCCAAT
COX2-R	CTAGGGAGGGGACTGCTCAT
mt-TFA-F	TTCCACAGAACAGCTACCCA
mt-TFA-R	CCCATCAGCTGACTTGGAGT
nuclear DNA-F	TGTTACCAACTGGGACGACA
nuclear DNA-R	CTATGGGAGAACGGCAGAAG
PAP-F	TGATCGAGACCCTCCAGC
PAP-R	CTCAATTACAGCTTGTGGAAGATTC
P450-F	CGTGGATGTGTTGACCCTCA
P450-R	CTCCACGTCTCTCAGCGAAA
U6-F	CTCGCTTCGGCAGCACA
U6-R	AACGCTTCACGAATTTGCGT
mmu-mir-125b-2-RT	GTCGTATCCAGTGCGTGTCGTGGAGTCGGCAATT
mmu-miR-125b-2-F	AGTCGTCCCTGAGACCCTAA
miR-unique-R	TAGTGCGTGTCGTGGAGT
Kif2b-F	CGGCAAGTTTTCCCTGGTTG
Kif2b-R	GCTGGCTCTGAATGGGGTAT
Gtsf11-F	CGTGGTTCCCATCAGAAAGC
Kif2b-R	AGGACGAACATTGGGTGACA
1700013D24Rik-F	TGGCATACCTTGGCTCTA
1700013D24Rik-R	ATCGGCACCTGAGAAAGA
Yod1-F	AGTCAGCGAATCCTCGTTGG
Yod1-R	AGACTCCTCCTTCCACGACA
Hils1-F	GTGGCTGGAGACCAAGAT
Hils1-R	TCAAGGTAGCAAGGGACA
GK2-F	CAACTCCACTCGCTTTCT
GK2-R	GCTCCACCCATCCTTCTT
Oser1-F	CTGTTCTTCATCCTCCCAAAT
Oser1-R	TCTCCTGCGTTGAACTCTTT
β-actin-F	TGCTGTCCCTGTATGCCTCT
β-actin-R	CTTTGATGTCACGCACGATTT
